# Cloning, Expression, and Characterization of Thermotolerant Manganese Superoxide Dismutase from *Bacillus* sp. MHS47

**DOI:** 10.3390/ijms12010844

**Published:** 2011-01-24

**Authors:** Supatra Areekit, Pornpimon Kanjanavas, Paisarn Khawsak, Arda Pakpitchareon, Kajeenart Potivejkul, Gaysorn Chansiri, Kosum Chansiri

**Affiliations:** 1 Department of Biochemistry, Faculty of Medicine, Srinakharinwirot University, Bangkok 10110, Thailand; E-Mails: jeedkha@hotmail.com (S.A.); pkhla@hotmail.com (P.K.); oom_arda@hotmail.com (A.P.); 2 Department of Biology, Faculty of Science and Technology, Rambhai Barni Rajabhat University, Chanthaburi 22000, Thailand; E-Mail: kanjanavas@hotmail.com; 3 Department of Biology, Faculty of Science, Srinakharinwirot University, Bangkok 10110, Thailand; E-Mail: kajeenart@gmail.com; 4 Department of Pharmaceutical Technology, Faculty of Pharmacy, Silpakorn University, Nakhon Pathom 73000, Thailand; E-Mail: gaysorn@email.pharm.su.ac.th

**Keywords:** thermotolerant, MnSOD, cloning, expression, *Bacillus* sp

## Abstract

A superoxide dismutase gene from thermotolerant *Bacillus* sp. MHS47 (*MnSOD47*) was cloned, sequenced, and expressed. The gene has an open reading frame of 612 bp, corresponding to 203 deduced amino acids, with high homology to the amino acid sequences of *B. thuringiensis* (accession no. EEN01322), *B. anthracis* (accession no. NP_846724), *B. cereus* (accession no. ZP_04187911), *B. weihenstephanensis* (accession no. YP_001646918), and *B. pseudomycoides*. The conserved manganese-binding sites (H28, H83, D165, and H169) show that MnSOD47 has the specific characteristics of the manganese superoxide dismutase (MnSOD) enzymes. *MnSOD47* expressed an enzyme with a molecular weight of approximately 22.65 kDa and a specific activity of 3537.75 U/mg. The enzyme is active in the pH range 7–8.5, with an optimum pH of 7.5, and at temperatures in the range 30–45 °C, with an optimum temperature of 37 °C. Tests of inhibitors and metal ions indicated that the enzyme activity is inhibited by sodium azide, but not by hydrogen peroxide or potassium cyanide. These data should benefit future studies of MnSODs in other microorganisms and the biotechnological production of MnSOD47, and could also be used to develop a biosensor for the detection of antioxidants and free radical activity. In the future, this basic knowledge could be applicable to the detection of cancer risks in humans and therapeutic treatments.

## 1. Introduction

Superoxide dismutase (SOD) is a metabolic enzyme that specifically catalyzes the conversion of the superoxide radical (O^2−^) to H_2_O_2_ and O_2_. SODs are considered key enzymes in the control of oxidative stress because they can protect oxygen-metabolizing cells against the harmful effects of superoxide free radicals [1–4]. The SOD metalloenzymes can be separated into three classes based on the metal cofactors at their active sites: copper/zinc SOD (Cu/ZnSOD), manganese SOD (MnSOD), and iron SOD (FeSOD) [5,6].

Recently, SODs have been used in gene therapy and therapeutic treatments for oxidative damage in the treatment of postischemic reperfusion injury, rheumatoid arthritis and osteoarthritis, brain trauma, influenza-induced lung pneumonitis, breast cancer, nervous system dysfunction, persistent pulmonary hypertension, and tissue damage. SODs are considered to be clinically useful for a wide variety of applications, including the prevention of oncogenesis, tumor promotion, and tumor invasiveness, and the reduction of the cytotoxic and cardiotoxic effects of anticancer drugs [7–17]. A SOD biosensor has also been used to determine the antioxidant properties of acetylsalicylic-acid-based drugs and the antiradical activity of healthy and cancerous human brain tissues [18].

Today, thermostable enzymes play important roles in industry because of their stability. Among these, thermostable SODs from thermotolerant or thermophilic microorganisms have received increasing attention [19]. SODs have been isolated from hyperthermophiles of the genera *Sulfolobus* and *Pyrobaculum*, and from *Aquifex pyrophilus*, *Thermothrix* sp., and *Rhodothermus* sp. [20–25].

In this study, we focused on the isolation and characterization of the gene encoding SOD in the thermotolerant microorganism *Bacillus* sp. MHS47, which was isolated from hot springs. The *SOD* gene and its expression were investigated. Our results should facilitate its use in pharmaceutical and medical research, and its biotechnological production.

## 2. Results and Discussion

### 2.1. Isolation of the *Bacillus* sp. MHS47

Soil and water samples from hot springs in Mae Hong Son Province of Thailand were previously collected and screened for thermotolerant bacteria using a dilution technique, and were cultured at 45 °C. Isolate MHS47 is a Gram-positive, rod-shaped aerobic bacterium. It was identified by 16S ribosomal DNA (rDNA) analysis and this sequence was deposited in GenBank under accession number HQ166833. Comparison of its nucleotide sequence revealed that the isolate was 99% homologous to *B. cereus*.

### 2.2. Expression and Purification of MnSOD47

**Figure 1:**
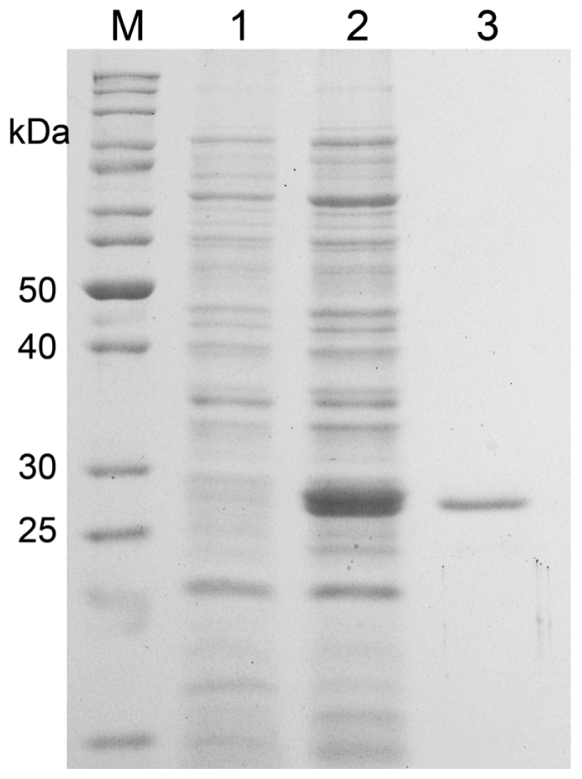
SDS–PAGE profile of the purified recombinant MnSOD47 enzyme. Lanes: M, standard protein marker; 1, MnSOD47 before induction with IPTG; 2, MnSOD47 after induction with 0.5 mM IPTG; 3, Purified MnSOD47. Visualized by Coomassie-Blue-staining.

The gene encoding MnSOD (*MnSOD47*) was amplified from the genomic DNA of *Bacillus* sp. MHS47, using primers designed to *Bacillus* spp. The *SOD* gene sequences have been submitted to GenBank, as indicated in the Experimental Section. PCR amplification generated a product of 612 bp. The fragment was ligated and cloned, and three positively transformed colonies were selected. The nucleotide sequence confirmed that the complete *MnSOD47* gene is 612 bp long (accession number HQ112282), corresponding to 204 deduced amino acids, with a molecular weight (MW) of approximately 22.65 kDa and a pI of 5.31 (Figure 1).

**Figure 2:**
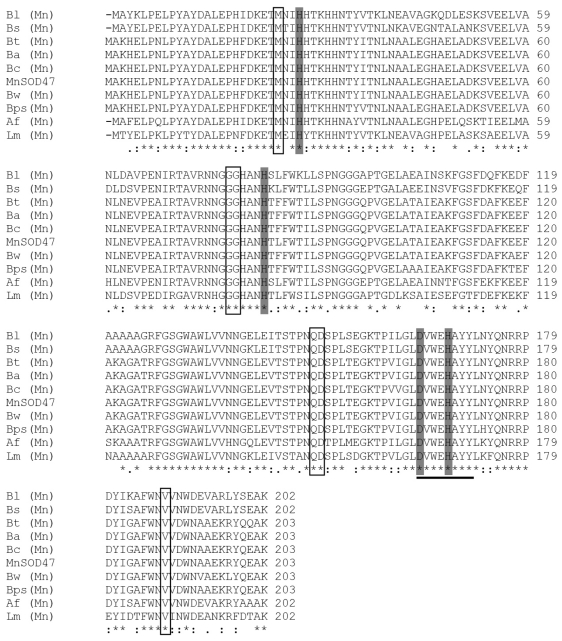
Alignment of the amino acid sequence of MnSOD47 with those of other bacterial MnSODs: *B. licheniformis* (Bl, accession no. YP_079829); *B. subtilis* (Bs, accession no. ZP_03592273); *B. thuringiensis* (Bt, accession no. EEN01322); *B. anthracis* (Ba, accession no. NP_846724); *B. cereus* (Bc, accession no. ZP_04187911); *B. weihenstephanensis* (Bw, accession no. YP_001646918); *B. pseudomycoides* (Bps, accession no. ZP_04152942); *Anoxybacillus flavithermus* (Af, accession no. YP_ 002315239); and *Listeria monocytogenes* (Lm, accession no. YP_ 079829). The residues highlighted in dark gray represent the four metal-binding sites, which coordinate the metal ion. These residues are conserved in all reported FeSODs and MnSODs. The amino acids characteristic of the MnSODs are boxed [28,29]. The MnSOD signature of the decapod crustaceans (DXWEHXXY) is underlined.

**Figure 3:**
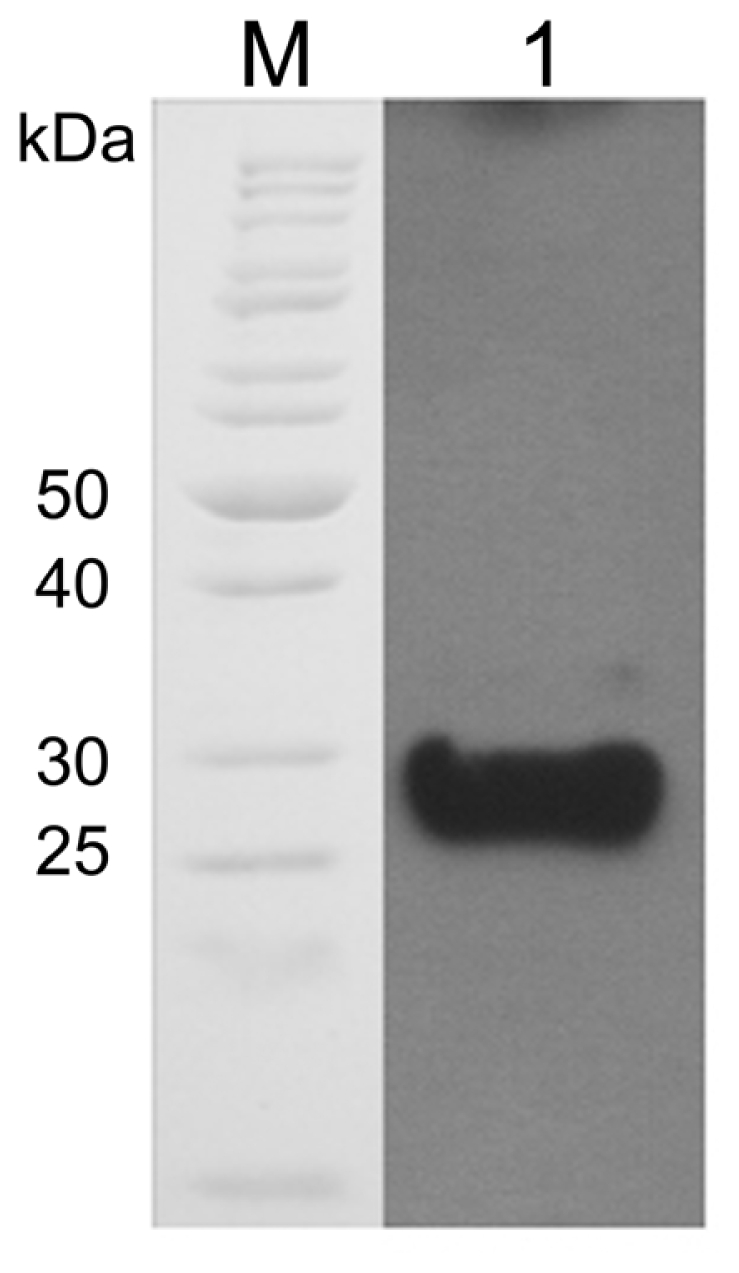
Western blot analysis of MnSOD probed with rabbit anti-Cu/Zn SOD antibody. Lanes: M, standard protein marker; 1, MnSOD.

An amino acid comparison of the active site and the conserved and semiconserved regions indicated that the MnSOD47 enzyme is similar to those regions of *B. cereus* AH1271, with 99% homology. The four residues of the enzyme that are putatively essential to coordinate the single trivalent manganese (H28, H83, D165, H169; Figure 2) are conserved, as they are similarly conserved in other reported MnSODs [26]. MnSOD47 also contains the decapod crustacean signature (DXWEHXXY), which is a specific characteristic of MnSOD [27]. Western blot analysis of MnSOD47 probed with rabbit anti-Cu/Zn SOD antibody confirmed that the enzyme is a SOD (Figure 3). The specific activity of the purified enzyme was 3537.75 U/mg, with a protein recovery of 54.3% and 14-fold purification (Table 1).

### 2.3. Effect of pH and Temperature on MnSOD47 Activity

**Figure 4:**
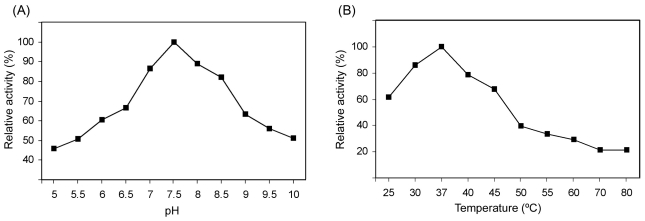
Effect of (**A**) pH, and (**B**) temperature on the enzymatic activity of MnSOD47.

**Figure 5:**
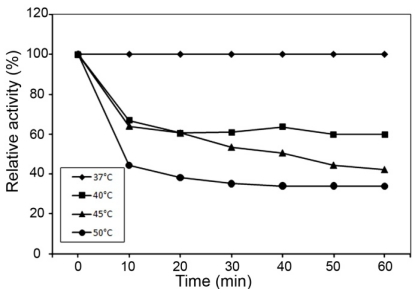
Thermostability of MnSOD47 activity.

The effect of pH and temperature on the enzyme activity of MnSOD47 and the kinetics of its thermostability are shown in Figure 4. The enzyme is active in the pH range 7–8.5, with an optimum at pH 7.5 (Figure 4A) and in the temperature range of 30–45 °C, with an optimum at 37 °C (Figure 4B). The temperature stability test showed that 50% activity was retained after its incubation at 45 °C for 40 min (Figure 5).

### 2.4. Effect of Inhibitors on MnSOD47 Activity

**Figure 6:**
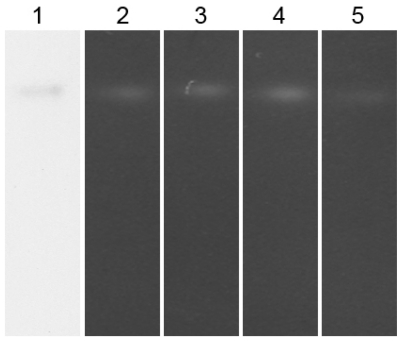
Activity staining of MnSOD47 in the presence of various inhibitors. The zymogram was produced with 12.5% native polyacrylamide gel electrophoresis. Lane 1: stained protein; lane 2: with no inhibitor; lane 3: with 10 mM KCN; lane 4: with 10 mM H_2_O_2_; lane 5: with 10 mM NaN_3_.

The enzyme’s activity was inhibited by sodium azide (NaN_3_), but not by hydrogen peroxide (H_2_O_2_) or potassium cyanide (KCN). Figure 6 shows the effects of the inhibitors on MnSOD47 activity, demonstrated with 12.5% native polyacrylamide gel electrophoresis. The inhibition of MnSOD47 activity in the presence of NaN_3_ indicates that the enzyme is MnSOD.

### 2.5. Discussion

In this study, the complete *MnSOD47* gene was cloned and its nucleotide sequence was analyzed. The 204 deduced amino acids corresponded to a calculated monomeric MW of 22.65 kDa, similar to those of the MnSODs in *B. thuringiensis* (accession no. EEN01322), *B. anthracis* (accession No. NP_846724), *B. cereus* (accession no. ZP_04187911), *B. weihenstephanensis* (accession No. YP_001646918), and *B. pseudomycoides* (accession no. ZP_04152942). The most frequently reported molecular weights for the MnSODs range from 20 to 40 kDa [30]. The theoretical isoelectric point (pI) of the protein was calculated to be 5.31, which is in the range of known SOD pI values of 4.0–6.0 [31]. SDS–PAGE analysis showed that the molecular mass of MnSOD47 was approximately 27 kDa, which is higher than the calculated molecular mass. This effect is attributed to the addition of a polyhistidine tag at the 5′ end of the recombinant sequence [32]. According to the characteristics of the MnSOD47 amino acid sequence, the enzyme shares the features conserved in the SODs, including the manganese-binding site, which consists of three histidines and one aspartate residue (H28, H83, H169, and D165) [26]. In addition to the metal ligands, a number of other residues are strictly conserved, including an aspartic acid residue (D165, according to MnSOD47 sequence numbering) that is part of the distinctive DXWEHXXY sequence motif, which contains two of the four protein-donated metal ligands (E168 and H169). The cysteine residues that are inferred to form an intrasubunit disulfide bridge in other SODs are absent. The specific activity of the purified enzyme is 3537.75 U/mg. This value is higher than those of the MnSOD activities reported for *Thermus aquaticus* [33] and *Haliotis discus* [31], but lower than the unusually stable MnSOD reported from *Tatumella ptyseos* [34]. The optimum pH and optimum temperature for MnSOD47 activity are similar to those of MnSOD from the disk abalone (*Haliotis discus discus*), which was cloned and expressed in *Escherichia coli* K12 (TB1) [31] and has an optimum temperature of 37 °C. The enzymatic activity of MnSOD47 is inhibited by NaN_3_, but not by H_2_O_2_ or KCN, indicating that the enzyme is MnSOD [1,19,35,36].

## 3. Experimental Section

### 3.1. Bacterial Strain Collection

A thermotolerant *Bacillus* was isolated from hot spring water in the Mae Hong Son Province of Thailand. Soil and water samples had previously been screened for bacterial strains using a dilution technique, cultured in Luria–Bertani (LB) broth at 45 °C for 24 h, and were then cross-streaked on LB agar. Pure single colonies were transferred to an LB slant for stock culture. The purified colony was identified using the API^®^ 50 CH Kit (BioMerieux, Lyon, France) and 16S rDNA nucleotide sequence analysis.

### 3.2. Genomic DNA Extraction

The selected Bacillus isolate with the highest production of SOD was cultured in LB medium at 45 °C for 12–14 h. The genomic DNA was extracted from *Bacillus* sp. MHS47 using a genomic DNA Purification Kit (Gentra Systems, Minneapolis, USA). The DNA pellet was dissolved in TE buffer and stored at 4 °C.

### 3.3. PCR Amplification of the 16S rDNA

PCR amplification was performed using specific universal primers for bacterial 16S rDNA (8F 5′-AGAGTTTGATCCTGGCTCAG-3′ and 1492R 5′-ACGGTTACCTTGTTACGACTT-3′) [37]. All the reactions were manipulated in 25 μL volumes containing 50 ng of genomic DNA in 10 × PCR buffer, 1 μM each primer, 100 μM dNTP, 1.5 mM MgCl_2_, and 1.5 U of proofreading *Taq* DNA polymerase (Invitrogen^®^). PCR was performed in a DNA thermal cycler (MJ Research PTC-200 Peltier) for 30 cycles. Each cycle consisted of denaturation at 94 °C for 1 min, annealing at 54 °C for 1 min, and extension at 72 °C for 1 min.

### 3.4. PCR Amplification of the *MnSOD* Gene from *Bacillus* sp. MHS47

The *MnSOD47* gene was PCR amplified using SOD primers designed for *Bacillus* spp. (accession numbers AE016879, AE017194, AE017225, AE017355, AE017334, CP000001, and CP000485). A CACC sequence was added to the 5′ end of the forward primer, followed by the nucleotide sequence specific for *MnSOD47*. The forward and reverse primers (SOD-BacillusF and SOD-BacillusR) were 5′-CACCATGGCAAAACACGAATT-3′ and 5′-TTATTTTGCTTCTTGGTAACG-3′, respectively. The set of primers was synthesized by Invitrogen^®^. All the reactions were performed in 25 μL volumes containing 100 ng of genomic DNA in 10 × PCR buffer, 20 μM each primer, 10 μM dNTPs, 1.5 mM MgCl_2_, and 1.5 U of proofreading *Taq* DNA polymerase (Invitrogen^®^). The PCR was performed using a DNA thermal cycler for 30 cycles. Each cycle consisted of denaturation at 94 °C for 1 min, annealing at 45 °C for 1 min, and extension at 72 °C for 1 min. The PCR fragment was analyzed by electrophoresis on a 1.2% agarose gel. The PCR product was eluted from the gel and purified using the QIAquick^®^ gel extraction kit (Qiagen, Hilden, Germany) before it was cloned.

### 3.5. Cloning the PCR Fragment

The purified PCR fragment was ligated to the pET 100/D TOPO^®^ vector using the Champion™ pET Directional TOPO^®^ Expression Kit (Invitrogen^®^). The reaction was performed according to the protocol provided by the manufacturer. White colonies containing recombinant DNA were selected and cultured in 5 mL of LB medium in the presence of 100 mg/L ampicillin for 24 h.

### 3.6. DNA Sequencing of the *SOD* Gene

Recombinant plasmids containing the *MnSOD47* gene were purified using the Qiagen^®^ QIAprep Spin Miniprep Kit and sequenced in both directions using the BigDye Terminator Cycle Sequencing Kit (Macrogen, Korea). The nucleotide and amino acid sequences were analyzed with the BLAST program at http://www.ncbi.nlm.nih.gov/BLAST.

### 3.7. Transformation and Expression of the *MnSOD47* Gene

The recombinant DNA was transformed into *E. coli* BL21 Star™ (DE3) One Shot^®^ cells. The transformation was performed according to the protocol provided by the manufacturer. The recombinant cells were grown in 500 mL of LB containing 50 μL/mL ampicillin and 1% glucose at 37 °C until an optical density of 0.5 at 600 nm was reached. IPTG was added to a final concentration of 1 mM and the samples were incubated for 6 h.

### 3.8. Purification of the MnSOD47 Enzyme

The MnSOD47 protein was purified as the polyhistidine-tagged recombinant protein on Protino^®^ Ni-TED resin (Macherey-Nagel, Germany) under nondenaturing conditions. The protocol used was provided by the manufacturer. The eluted fractions were stored at 4 °C before their analysis by SDS-PAGE.

### 3.9. SDS-PAGE

Traditional SDS-PAGE was performed on a 12.5% separating gel to confirm the purified and apparent MW of the SOD enzyme, with Tris-glycine (pH 8.3) as the buffer [38].

### 3.10. Protein Assay

Protein concentrations were determined with the Bradford Protein Assay (Fermentas, USA), using bovine serum albumin as the standard, with the absorbance measured at 595 nm.

### 3.11. Immunoblotting Analysis

Immunoblotting analysis was performed with the ECL Western blotting analysis system (Santa Cruz Biotechnology). SDS-PAGE was performed as described above. The proteins were blotted onto a sheet of polyvinylidene difluoride transfer membrane (Pall Life Science, Pensacola, FL), according to the instructions of the manufacturer. After blotting, the membrane was blocked by incubation with 5% skim milk and PBS buffer (pH 7.5) for 2 h at room temperature. The blocked membrane was incubated at 4 °C for a further 12 h with primary anti-SOD1 antibody (rabbit polyclonal; 1:1000 dilution) before it was washed in TPBS (PBS buffer [pH 7.5] and 0.15% Tween 20) for 1 h at room temperature. The membrane was then incubated with horseradish peroxidase (HRP)-conjugated goat anti-rabbit IgG antibody and the HRP–streptavidin complex for 1 h at room temperature. After repeated washing with TPBS buffer for 1 h at 4 °C, the membrane was incubated with ECL detection reagent (SuperSignal West Pico, Rockfort, USA), before visualization with autoradiography.

### 3.12. Enzyme Assay

The activity of the purified recombinant MnSOD47 was determined with a Chemical SOD Assay Kit (Cayman, USA). The absorbance was measured at 450 nm with a spectrophotometer, using a xanthine oxidase/hypoxanthine generating system coupled to a tetrazolium salt to detect the reduction in superoxide radicals.

### 3.13. Effect of pH and Temperature on MnSOD47 Activity

The optimum pH was determined in the presence of various buffer systems in the pH range of 5–10. The buffers used were: 0.1 M CH_3_COOH–CH_3_COONa (pHs 5.0, 5.5, 6.0), 0.1 M NaH_2_PO_4_–Na_2_HPO_4_ (pHs 6.0, 6.5, 7.0, 7.5), 0.1 M Tris-HCl (pHs 7.5, 8.0, 8.5, 9.0), and 0.1 M glycine–NaOH (pHs 9.0, 9.5, 10.0). MnSOD47 activity was determined as the percentage residual activity relative to that of the control. The optimum temperature was analyzed in the range of 25–80 °C under standard conditions.

### 3.14. Thermostability

The thermal stability of MnSOD47 was examined in the temperature range of 37–50 °C. The enzyme was incubated in 0.1 M NaH_2_PO_4_-Na_2_HPO_4_ (pH 7.5) buffer, and samples were removed at fixed time intervals. The reactions were allowed to cool on ice before the residual activity was determined under standard conditions. MnSOD47 activity was determined as the percentage residual activity relative to that of the control.

### 3.15. Enzyme Inhibitors

Final 10 mM concentrations of chemical reagents such as KCN, H_2_O_2_, and NaN_3_ were used as inhibitors of MnSOD activity. The enzyme was preincubated with each inhibitor at pH 7.5 and 37 °C for 1 h, before electrophoresis on a 12.5% native gel, to determine the MnSOD activity. The residual activity of each preincubated sample was measured using the NBT method for parallel comparisons [39].

## 4. Conclusion

In conclusion, we cloned the gene encoding MnSOD from *Bacillus* sp. MHS47 and characterized the recombinant MnSOD protein expressed in *E. coli* cells. The deduced amino acid sequence showed high identity (99%) to the sequence of MnSOD from *Bacillus cereus*, and its biochemical properties are similar to those of other known MnSODs. Based on these sequence and biochemical analyses, we conclude that the putative *MnSOD47* clone encodes a cellular MnSOD. This information on MnSOD47 may be useful in studies of the MnSODs of other thermotolerant microorganisms and in its biotechnological production. These data could be used to develop a biosensor for the detection of antioxidants and free radical activity. In the future, this basic knowledge could also be used to assess cancer risk in humans, and in therapeutic treatments.

## Figures and Tables

**Table 1:** Purification of MnSOD47.

Purification	Total Volume (mL)	Total Protein (mg)	Total Activity (U)	Specific Activity (U/mg)	Protein Recovery (%)	Purification (fold)
Crude Extract Non-Induce	25	15.31	1701	111.10	-	-
Crude Extract Induce	25	14.77	3648	252.13	100	1
Ni-NTA	4.5	0.56	1981	3537.75	54.3	14
